# Oseltamivir-resistant influenza A(H1N1)pdm2009 strains found in Brazil are endowed with permissive mutations, which compensate the loss of fitness imposed by antiviral resistance

**DOI:** 10.1590/0074-02760140330

**Published:** 2015-02

**Authors:** Thiago Moreno Lopes e Souza, Natalia Fintelman-Rodrigues, Paola Cristina Resende, Milene Mesquita, Tatiana Schaffer Gregianini, Fernando A Bozza, Ana Carla Pecego, Sandra Bianchini Fernandes, Ana Luisa Furtado Cury, Irina Nastassja Riediger, Marilda M Siqueira

**Affiliations:** 1 Laboratório de Vírus Respiratórios e do Sarampo; 2 Laboratório de Imunofarmacologia, Instituto Oswaldo Cruz; 3 Centro de Desenvolvimento Tecnológico em Saúde, Fundação Oswaldo Cruz, Rio de Janeiro, RJ, Brasil; 4 Laboratório Central de Saúde Pública do Estado do Rio de Grande do Sul, Seção de Virologia, Fundação Estadual de Produção e Pesquisa em Saúde, Porto Alegre, RS, Brasil; 5 Laboratório de Medicina Intensiva, Instituto de Pesquisas Clínicas Evandro Chagas; 6 Laboratório Central de Saúde Pública do Estado de Santa Catarina, Florianópolis, SC, Brasil; 7 Laboratório Central de Saúde Pública do Estado de Minas Gerais, Instituto Octávio Magalhães, Fundação Ezequiel Dias, Belo Horizonte, MG, Brasil; 8 Laboratório Central de Saúde Pública do Estado do Paraná, Curitiba, PR, Brasil

**Keywords:** influenza A(H1N1)pdm09, oseltamivir, antiviral, resistance, H275Y, permissive mutation

## Abstract

The 2009 pandemic influenza A virus outbreak led to the systematic use of the neuraminidase (NA) inhibitor oseltamivir (OST). Consequently, OST-resistant strains, carrying the mutation H275Y, emerged in the years after the pandemics, with a prevalence of 1-2%. Currently, OST-resistant strains have been found in community settings, in untreated individuals. To spread in community settings, H275Y mutants must contain additional mutations, collectively called permissive mutations. We display the permissive mutations in NA of OST-resistant A(H1N1)pdm09 virus found in Brazilian community settings. The NAs from 2013 are phylogenetically distinct from those of 2012, indicating a tendency of positive selection of NAs with better fitness. Some previously predicted permissive mutations, such as V241I and N369K, found in different countries, were also detected in Brazil. Importantly, the change D344N, also predicted to compensate loss of fitness imposed by H275Y mutation, was found in Brazil, but not in other countries in 2013. Our results reinforce the notion that OST-resistant A(H1N1)pdm09 strains with compensatory mutations may arise in an independent fashion, with samples being identified in different states of Brazil and in different countries. Systematic circulation of these viral strains may jeopardise the use of the first line of anti-influenza drugs in the future.

Anti-influenza drugs are essential for prophylactic and therapeutic interventions. Since antiviral resistance to adamantanes is very common (Santesso et al. 2013), neuraminidase inhibitors (NAI) have become the main class of anti-influenza drugs in clinical use. Among NAIs, oseltamivir (OST) use has grown since the emergence of 2009 pandemic influenza A [A(H1N1)pdm09]. Consequently, the pressure imposed by OST on A(H1N1)pdm09 has led to the selection of OST-resistant mutants, with a prevalence of 1-2% in different countries (Dixit et al. 2013). Remarkably, community transmission of OST-resistant influenza A(H1N1)pdm09 has drawn special attention because mutation H275Y in the neuraminidase (NA) may decrease viral fitness (Kelso & Hurt 2012 ). Nevertheless, the H275Y change may emerge in a NA endowed with permissive mutations (Kelso & Hurt 2012), which compensates for the decrease in viral fitness imposed by OST resistance. Viral strains carrying predicted permissive mutations have been circulating (Hurt et al. 2011, 2012, Kelso & Hurt 2012, CDC 2013, Takashita et al. 2014, Zaraket et al. 2014, Correia et al. 2015) and may have been responsible for A(H1N1)pdm09 OST-resistant outbreaks in community settings (Hurt et al. 2011, 2012, Lackenby et al. 2011, Kelso & Hurt 2012, Storms et al. 2012, CDC 2013, Souza et al. 2013, Takashita et al. 2014, Zaraket et al. 2014, Correia et al. 2015). The largest cluster of community spread of OST-resistant A(H1N1)pdm09 occurred in Australia, with cases detected in cities 4,000 km apart (Hurt et al. 2011, 2012). In Brazil during 2012, we found an overall incidence of 1.19% of OST-resistant strains of influenza A(H1N1)pdm09 (Souza et al. 2013), with community spread in cities 535 km apart. In the current study, 2013 influenza A(H1N1)pdm09 surveillance data reveals the circulation of OST-resistant strains with predicted permissive mutations, detected by Sanger sequencing, in community settings in Brazil.

## SUBJECTS, MATERIALS AND METHODS

*Ethics *- Since influenza surveillance is covered by Brazilian public health policies and all data were analysed in an anonymous fashion, ethical committee approval and need for informed consent have been waived, as previously described (Souza et al. 2013).

*Patients and data collection* - A sub-set of samples from patients displaying acute symptoms of respiratory infection (fever, > 37.8ºC and respiratory influenza-like illness) (WHO 2011 ) were collected and sent to the National Influenza Centre (NIC) in Brazil. Patients were treated according to Brazilian guidelines for influenza management (MS 2009). Patients' information, such as name initials, gender, age, city/state of onset of illness and the dates of the beginning of the symptoms and sample collection, were registered.

*Sample collection and diagnosis* - Nasopharyngeal aspirates or Dacron swabs were collected and RNA was extracted using a viral RNA mini kit (QIAGEN, USA), according to the manufacturer's instructions. One-step real-time reverse transcription-polymerase chain reaction (RT-PCR) assays for influenza subtyping were performed according to the World Health Organization (WHO) recommendations (WHO 2011).

*Cells and virus isolation* - Madin-Darby canine kidney cells (London line) were kindly donated by the Centers of Disease Control and Prevention (CDC), Influenza Reagent Resources (FR-58). These cells were cultured in Dulbecco's modified Eagle's medium (GIBCO, USA) supplemented with 10% foetal bovine serum (Hyclone, USA), 100 U/mL penicillin and 100 µg/mL streptomycin and were incubated at 37ºC in 5% CO_2_ (WHO 2011). The virus isolation was done according to WHO international protocol (WHO 2011). We confirmed viral isolation by NA activity (Szretter et al. 2006, WHO 2009, 2011). Viruses were passaged no more than two times.

*Functional antiviral assay* - To determine the half maximum inhibitory concentration (IC_50_) values of our samples to OST carboxylate, we performed functional antiviral assays using the NA-Star^TM^ assay kit (Life Technologies, USA), according to the manufacturer's instructions (Souza et al. 2013). Assays with wild-type and resistant strains of influenza A(H1N1)pdm09, A/Perth/265/2009 and A/Perth/261/2009, respectively, were performed as a control. These control strains were kindly donated by International Society for Influenza and other Respiratory Viruses Diseases-Antiviral Group, Neuraminidase Inhibitor Susceptibility Network.

*Molecular antiviral assays* - Single nucleotide polymorphisms in the NA gene were analysed by pyrosequencing, as described previously (Deyde et al. 2010).

The NA gene was sequenced by Sanger sequencing according to a protocol described elsewhere (Baillie et al. 2012). The amplified RT-PCR products were purified using the QIAquick PCR Purification kit (QIAGEN) and sequenced using a BigDye Terminator v.3.1 Cycle Sequencing kit (Life Technologies). The products were analysed in an ABI Prism 3130XL genetic analyser (Life Technologies). Sequences with the mutation H275Y found in our analysis were deposited in GenBank (accessions KC984901, KC984933, KJ493404 and KJ493405). The data generated were assembled in Sequencher 5.0 software (GeneCodes Corporation, USA) with an NA reference sequence, A/California/4/2009 (GenBank accession FJ966084). N1 numbering was used for NA throughout this study.

## RESULTS AND DISCUSSION

In 2013, the Brazilian NIC received 1,498 specimens from individuals with acute respiratory infection, encompassing samples from three out of five Brazilian Regions. Among these, 310 were positive for influenza virus A(H1N1)pdm09. Most of the cases were concentrated in the southern (52.9%) and southeastern (31.3%) regions of Brazil. The analysed samples were collected mainly during autumn (28.4%) and winter (36.1%). Based on clinical-epidemiological forms completed, symptoms of severe acute respiratory infection were found in 2% of the patients, vaccines accounted for 13.5% of the individuals, comorbidities were registered in 13.9% of the patients and 7.7% deceased. OST-treated patients accounted for 15.9% of the individuals analysed. Among all confirmed cases of A(H1N1)pdm09, 208 samples presented reliable pyrograms, with respect to screening for the H275Y mutation (Deyde et al. 2010). Two specimens contained A(H1N1)pdm09 virus with the H275Y amino acid substitution indicative for OST resistance were found (Table). These were from individuals with no registered use of OST (Table), as occurred in 2012 (Souza et al. 2013). The IC_50_ values for sensitive strains (n = 206) isolated in 2013 were 0.5 ± 0.4 nM (mean ± standard deviation) and the IC_50_ values for resistant strains were 102 and 116 nM (Table).

**TABLE:** Viral characteristics and clinical aspects of patients in which oseltamivir (OST)-resistant samples were detected

Patients	Mutations	IC_50_(nM)	Region	State	Age	Gender	Symptoms onset(date)	Sample collection(date)	OST use	Deceased
1	H275Y	102	Southeast	RJ	40	F	2 April 2013	5 April 2013	No	No
2	H275Y	116	South	RS	26	F	26 March 2013	5 April 2013	No	No

IC50: the half maximum inhibitory concentration; OST: oseltamivir; RJ: Rio de Janeiro; RS: Rio Grande do Sul.

**Fig. 1:**
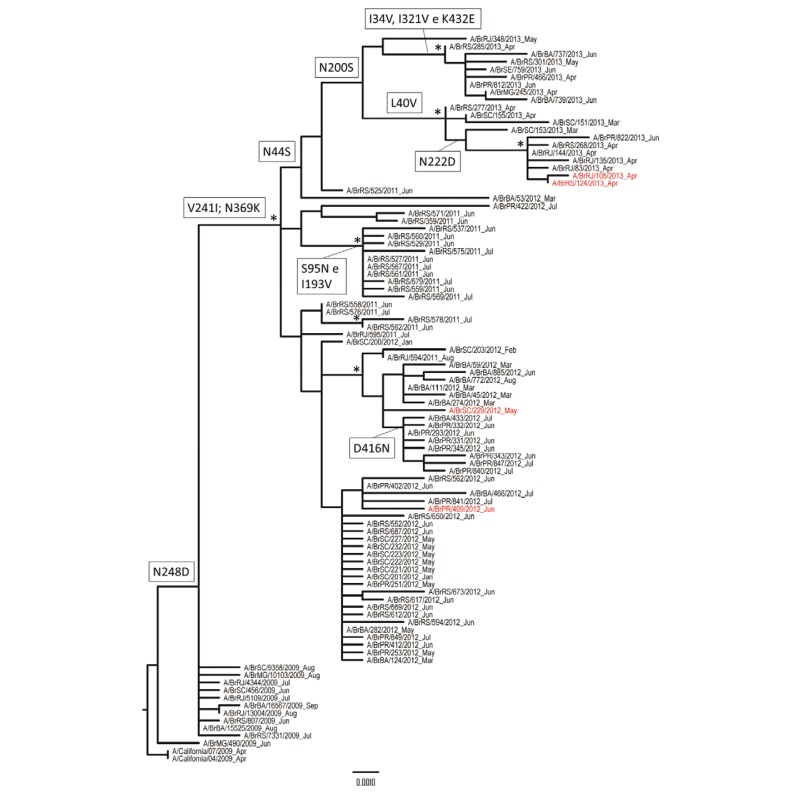
phylogenetic tree neuraminidase (NA) sequences from Brazil. Maximum-likelihood (ML) phylogenetic tree with NA from influenza A(H1N1)pdm09 viruses circulating in Brazil from 2009-2013, rooted by NA sequences from A/California/4/2009 and A/California/7/2009. Sequences with the mutation H275Y are shown in red. Amino acid changes associated with these specific clusters are indicated in the nodes using N1 numbering. ML reliability of branches was evaluated using approximate likelihood-ratio test and the interior branch cut-off values ≥ 0.9 are represented by asterisks.

In general, viral strains found in Brazilian community settings from 2013 clustered in a different branch than those from 2012 (Fig. 1) (H275Y viruses in red). This temporal segregation of NA sequences may suggest a tendency of positive selection of A(H1N1)pdm09 NAs. It is therefore likely that viruses with these NAs are better adapted to propagate in their hosts than the predecessor strains. The NA from Brazilian strains from 2012 and 2013 had the changes V241I and N369K in common. These mutations have been found in Brazil since 2011 (Fig. 1) and are predicted to compensate for the negative effects of H275Y change (Hurt et al. 2012, Butler et al. 2014). The V241I and N369K enhance NA expression and activity in in vitro studies and this effect improve A(H1N1)pdm09 fitness (Butler et al. 2014). These two changes have also been found in the community cluster of OST-resistant A(H1N1)pdm09 in the United States of America (USA), Australia and Japan (Hurt et al. 2012, CDC 2013, Takashita et al. 2014). The permissive mutation N386K, on the other hand, was found in Australia and Japan (Hurt et al. 2012, Takashita et al. 2014), but not in the Americas (neither in USA or Brazil). Differently than Australian samples, Japanese, North American and Brazilian samples from 2013 had the change N200S (Storms et al. 2012, Takashita et al. 2014). Remarkably, one Brazilian sample from 2013 had the change D344N, which is predicted to occur in A(H1N1)pdm09 NA by *in silico* data and capable of compensating for the reduction in NA activity by H275Y (Bloom et al. 2011). In addition, other changes of apparently minor importance occurred in the NA sequences shown in red in the phylogenetic tree (Fig. 1) (GenBank accessions KC984901, KC984933, KJ493404 and KJ493405). For example, the mutations N42S (2012) and N44S (2013) may create new glycosylation sites (Hurt et al. 2012). Moreover, we also found mutations in the A(H1N1)pdm09 NA close to their equivalent amino acid residues in the widely disseminated OST-resistant seasonal H1N1 (Meijer et al. 2007, Hurt et al. 2009, 2012); such as S79P, I188T and N225D.

**Fig. 2:**
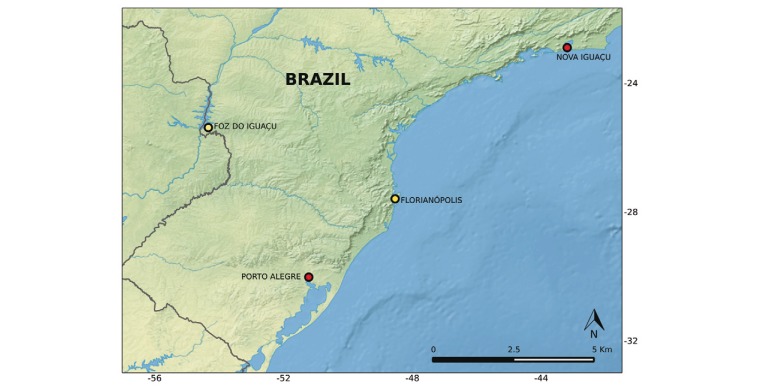
geographic detection of oseltamivir (OST)-resistant influenza A(H1N1)pdm09 strains in community settings. This map shows the Southern Cone of Brazil and neighbour Latin American countries. OST-resistant influenza A(H1N1)pdm09 detected in community settings in 2012 and 2013 are highlighted in red and yellow, respectively. This map has been generated with the R-program.

For public health concerns, H275Y viruses from 2012 were found in the cities of Foz do Iguaçu (state of Paraná) and Florianópolis (state of Santa Catarina) (Souza et al. 2013), whereas the two samples from 2013 were collected in Nova Iguaçu (NIG) (state of Rio de Janeiro) and Porto Alegre (POA) (state of Rio Grande do Sul) (Fig. 2). NIG and POA are cities around 1,490 km apart and have population densities of 1,527.60 and 2,837.52 inhabitant/km^2 ^(IBGE 2010), respectively. High population densities, such as these, increase the risk of respiratory virus infection and highlight the potential to spread OST-resistant variants. Considering the data from 2012 and 2013, an area of over 271,817 km^2 ^could be exposed to OST-resistant A(H1N1)pdm09 found in community settings. Remarkably, this area is within the southern and southeastern Brazilian regions, in which influenza activities are higher due to transition from a temperate to tropical climate.

Our data not only suggests that OST-resistant strains may be present in an even broader area of Brazil or South America, than previously thought (Souza et al. 2013), but also draws special attention to the community detection of influenza A(H1N1)pdm09 H275Y in highly populous developing countries in which antiviral resistance surveillance may be neglected. Although low prevalence of H275Y viruses with permissive mutations in the NA is comparable to what is found in other countries, this information is critical for further drug stockpiling and pandemic preparedness.
